# Two successful pregnancies in a woman with chronic myeloid leukemia exposed to nilotinib during the first trimester of her second pregnancy: case study

**DOI:** 10.1186/1756-8722-2-42

**Published:** 2009-10-06

**Authors:** Monika Conchon, Sabri S Sanabani, Israel Bendit, Fernanda Maria Santos, Mariana Serpa, Pedro Enrique Dorliac-Llacer

**Affiliations:** 1 Department of Hematology, Faculty of Medicine, University of São Paulo, São Paulo, Brazil; 2 Fundação Pro-Sangue, Hemocentro de São Paulo, Brazil

## Abstract

The occurrence of chronic myeloid leukemia in pregnancy is rare and its management poses a clinical challenge for physicians treating these patients. We report a 30-year-old woman with chronic myeloid leukemia who became pregnant twice successfully. Philadelphia-positive CML in its chronic phase was diagnosed at 16 weeks of her first gestation. At that time, she received no treatment throughout her pregnancy. At 38 weeks of gestation, a normal infant was delivered by cesarean section. At six weeks postpartum, the patient underwent imatinib mesylate therapy but she could not tolerate the treatment. The treatment was then changed to nilotinib at 400 mg orally b.i.d. Two years later, she became pregnant again while she was on nilotinib 200 mg b.i.d. The unplanned pregnancy was identified during her 7.4 weeks of gestation. Because the patient elected to continue her pregnancy, nilotinib was stopped immediately, and no further treatment was given until delivery. Neither obstetrical complications nor structural malformations in neonates in both pregnancies were observed. Both babies' growth and development have been normal. Although this experience is limited to a single patient, the success of this patient demonstrates that the management of chronic myeloid leukemia in pregnant women may be individualized based on the relative risks and benefits of the patient and fetus.

## Introduction

Chronic myeloid leukemia (CML) is a clonal myeloproliferative disorder that occurs as a result of a reciprocal translocation between chromosome 22 and chromosome 9, or the Philadelphia translocation [1]. This translocation creates a fusion gene-breakpoint cluster region *bcr-abl *proto-oncogene, which encodes an oncoprotein that has constitutively active *abl *tyrosine kinase (TK) activity. The introduction of the TK inhibitor (TKI) imatinib in 1998 indisputably advanced the clinical management of cancer. Imatinib has demonstrated its efficacy by increasing overall survival and substantially improving the life expectancy and quality of life of patients with CML. As a result, patients who are of childbearing age and are currently being treated with imatinib now find themselves contemplating reproductive opportunities that would not have otherwise been possible. However, the occurrence of CML during pregnancy poses a unique clinical challenge for physicians treating these patients and requires balancing concerns between maternal survival and fetal health in both the short- and long-term. Because imatinib was teratogenic in rats, it was strongly advised that effective contraception be used during therapy to prevent pregnancy [2]. The inability of patients to tolerate treatment and the emergence of *bcr-abl *mutations that reduced the binding affinity of imatinib prompted pharmaceutical research that led to the discovery of similarly effective, targeted, second generation TKIs such as nilotinib (Novartis) and dasatinib (Bristol-Myers Squibb). There is still insufficient efficacy and safety data on these newer medications to warrant their safety in pregnant women with CML. In this study, we are reporting the outcome of a patient with CML who became pregnant twice successfully. The patient was only observed without active intervention for the duration of her first pregnancy while received nilotinib at the time of her second conception.

## Case description

In February 2006, during a routine antenatal examination, a 30-year-old woman at 16 weeks of gestation was diagnosed with Philadelphia-positive CML in its chronic phase. She was normal upon physical examination without any noteworthy clinical symptoms. Laboratory studies showed hemoglobin 10.7 g/dL, platelets 101 × 10^9 ^L and white cell count 23,4 × 10^9 ^L with a differential revealing 7% myelocytes, 2% promyelocytes, 15% metamyelocytes, 28% band cells, 39% neutrophils, 3% eosinophils, 0% basophils, 4% lymphocytes and 2% monocytes. The diagnosis of CML was confirmed based on *bcr-abl *mRNA transcript detection and conventional chromosome banding, which revealed a 46,XY, t(9;22) karyotype. The patient was treated with 3 million IU × 5 of interferon alfa-2a every week throughout her pregnancy. At 38 weeks of gestation, a normal infant was delivered by cesarean section. In March 2007, she started imatinib 400 mg daily and continued oral contraceptives. In May 2007, while in complete hematological remission (HR), she developed grade 3 hepatotoxicity (aspartate aminotransferase (340 U/l: normal range (N) <40 U/l), alanine aminotransferase (640 U/l: *N *<40 U/l), gamma glutamyltransferase (423 U/l: *N *<80 U/l), total bilirubin (0.55 mg/dl) and alkaline phosphatase were normal) according to the NCI common toxicity criteria for adverse events (CTCAE v3.0). A serologic investigation for hepatitis A, B and C was negative. Because of hepatotoxicity, imatinib was temporary stopped, and oral contraceptives were discontinued. In the 4 weeks after imatinib was stopped, hepatotoxicity was reduced to grade 1. Another attempt at low dose imatinib (300 mg daily) was again followed by elevations in hepatic enzymes. Liver biopsy at the time showed histological evidence of subacute, drug-induced liver damage. Therefore, imatinib was permanently withdrawn. Consequently, the patient was treated with interferon alfa-2a at a dose of 9 million IU daily. After 1 year of IFN-based therapy, complete HR was achieved without any cytogenetic response. In April 2008, she entered a phase II trial testing nilotinib at 400 mg orally b.i.d. (CAMN07A2109). One week later, she developed grade 1 hepatotoxicity and grade 3 myelotoxicity evidenced by neutropenia with a neutrophil count of 0.78 × 10^9^l. According to the therapeutic protocol, the nilotinib dose was reduced to 200 mg b.i.d.

In August 2008, she was in complete cytogenetic remission and major molecular response. At that time, she became pregnant while she was on nilotinib 200 mg b.i.d. The unplanned pregnancy was identified during her first trimester of gestation after the patient had experienced 7.4 weeks of amenorrhea. The patient was informed of the potential fetal toxicities of therapy. After detailed and meticulous counseling, the patient elected to continue her pregnancy, so nilotinib was stopped immediately, and no further treatment was given until delivery. A follow-up with ultrasound scans during the course of the pregnancy was unremarkable. In April 2009, she delivered via cesarean section a healthy male baby weighing 3.2 kg with an Apgar score of 9 at 10 minutes at gestational week 33. He was breast-fed for 2 months. At 5 months post-partum, the patient's child has been healthy and developing normally. After delivery, the patient lost molecular, cytogenetic and HR. Because nilotinib was not affordable, the patient started dasatinib 100 mg daily in June 2009. She is currently in complete HR.

## Discussion

CML comprises less than 10% of leukemias in pregnancy and is very rare during conception; the incidence has been estimated as 1 per 100,000 pregnancies annually [3]. Over the last few decades, treatment of CML in pregnancy has consisted of conservative management with varying degrees of success including leukapheresis [4,5], hydroxyurea [6,7] and interferon [8-10]. The therapeutic management of CML in pregnant women with targeted therapies often presents divisive dilemmas and poses substantial challenges to both patients and their physicians. The key question to consider is whether to stay on these agents, which carry the risks of birth defects, or stop the medications and risk relapse. Although most of the existing data on the effects of imatinib on pregnancy have shown satisfactory outcomes, they do not indicate that it can be safely recommended during the first trimester of gestation [11-14]. One of the most comprehensive data sets on the effect of imatinib on pregnancy was recently reported by Pye and colleagues [15]. In Pye et al.'s study, imatinib was evaluated in 180 women who were exposed to treatment during pregnancy; outcomes were available for 125 patients. In total, 50% delivered a healthy baby, 28% elected to have a termination and 14% had a miscarriage. Twelve pregnancies resulted in infants with fetal abnormalities; 3 of which had strikingly similar complex malformations. In regards to the second generation TKIs, a literature search revealed only one report on the use of dasatinib during pregnancy [16]. Nilotinib, a potent TKI, was introduced in November 2007 for the treatment of patients with chronic or acute phase CML who were resistant to or intolerant of imatinib. Evidence from an in-vitro study indicated that nilotinib was 20 times more potent than imatinib against cells expressing the wild-type *bcr-abl *[17]. Compared to imatinib, nilotinib also has a higher binding affinity and selectivity for the inactive *abl *kinase conformation. Currently, nilotinib is classified as US Food and Drug Administration Pregnancy Category D with studies in rabbits showing it is associated with mortality, abortion and decreased gestational weights at a dose of 300 mg/kg/d (approximately half of the exposure used in humans based on area-under-curve (AUC)) with an overall lack of data in humans [18]. Based on the medical information provided by the manufacturer, nilotinib is not considered teratogenic, but an increased risk of embryo toxicity was noted at even sub-therapeutic doses. Similar to other TKIs, the manufacturer states that nilotinib is contraindicated during conception because of concerns it may cause fetal deformities. However, the medication safety information of nilotinib in pregnancy is obtained through animal studies, which may not apply to humans. A review of the literature did not identify any published data in women with CML who were treated with nilotinib when they conceived. In our patient, the first fetus had not been exposed to any therapy throughout the first pregnancy while the second fetus had been exposed to nilotinib during embryogenesis (weeks 8 to 12 of gestation) for approximately 8 weeks after the second conception. The outcome of the first pregnancy in our case was similar to that described by Cole et al [19] who reported on a 21-year-old woman with CML who was monitored without active intervention throughout her pregnancy with a favorable outcome for the patient and her infant. We found no published studies on the use of nilotinib during pregnancy, apart from the case report described above. In our case, pregnancy progressed normally and both infants were delivered at term without complications. There was no congenital anomalies and no late adverse effect, the older being now 3.4 years old and the younger 6 months old.

Although nilotinib treatment did not have a negative impact on this patient and her fetus during the second gestation, patients receiving nilotinib should be advised to practice adequate contraception. If the patient becomes pregnant while receiving the drug, the patient should be advised of the potential hazard to the fetus, and the drug should be discontinued.

## Conclusion

Although this experience is limited to a single patient, the success of this patient demonstrates that the management of CML in pregnant women may be individualized based on the relative risks and benefits of the patient and fetus and that the patient must be involved in decisions.

## Competing interests

The authors declare that they have no competing interests.

## Authors' contributions

MC was responsible of the clinical management of the patient, acquisition of data, drafting the manuscript; SS was responsible of the scientific revision, discussion and editing of the manuscript; IB, FMS, MS were involved in clinical management of the patient and interpretation of data, PED was supervisor of clinical management of the patient and interpretation of data. All authors read and approved the final manuscript.

## Consent

Written informed consent was obtained from the patient for publication of this case report. A copy of the written consent is available for review by the Editor-in-Chief of this journal.
